# Micro-patterned surfaces reduce bacterial colonization and biofilm formation *in vitro*: Potential for enhancing endotracheal tube designs

**DOI:** 10.1186/2001-1326-3-8

**Published:** 2014-04-16

**Authors:** Rhea M May, Matthew G Hoffman, Melinda J Sogo, Albert E Parker, George A O’Toole, Anthony B Brennan, Shravanthi T Reddy

**Affiliations:** 1 Sharklet Technologies, Inc., Aurora, 12635 E. Montview Blvd. Suite 155, CO 80045 Aurora, CO USA; 2 Center for Biofilm Engineering, and the Department of Mathematical Sciences, Montana State University, Bozeman, MT, USA; 3 Geisel School of Medicine at Dartmouth, Hanover, NH, USA; 4 Department of Materials Science and Engineering, University of Florida, Gainesville, FL, USA

**Keywords:** Micro-pattern, Sharklet, VAP, Endotracheal tube, Biofilm inhibition

## Abstract

**Background:**

Ventilator-associated pneumonia (VAP) is a leading hospital acquired infection in intensive care units despite improved patient care practices and advancements in endotracheal tube (ETT) designs. The ETT provides a conduit for bacterial access to the lower respiratory tract and a substratum for biofilm formation, both of which lead to VAP. A novel microscopic ordered surface topography, the Sharklet micro-pattern, has been shown to decrease surface attachment of numerous microorganisms, and may provide an alternative strategy for VAP prevention if included on the surface of an ETT. To evaluate the feasibility of this micro-pattern for this application, the microbial range of performance was investigated in addition to biofilm studies with and without a mucin-rich medium to simulate the tracheal environment *in vitro*.

**Methods:**

The top five pathogens associated with ETT-related pneumonia, Methicillin-Resistant *Staphylococcus aureus* (MRSA)*, Pseudomonas aeruginosa*, *Klebsiella pneumonia, Acinetobacter baumannii,* and *Escherichia coli,* were evaluated for attachment to micro-patterned and un-patterned silicone surfaces in a short-term colonization assay. Two key pathogens, MRSA and *Pseudomonas aeruginosa,* were evaluated for biofilm formation in a nutrient rich broth for four days and minimal media for 24 hours, respectively, on each surface type. *P. aeruginosa* was further evaluated for biofilm formation on each surface type in a mucin-modified medium mimicking tracheal mucosal secretions. Results are reported as percent reductions and significance is based on *t*-tests and ANOVA models of log reductions. All experiments were replicated at least three times.

**Results:**

Micro-patterned surfaces demonstrated reductions in microbial colonization for a broad range of species, with up to 99.9% (*p* < 0.05) reduction compared to un-patterned controls. Biofilm formation was also reduced, with 67% (*p* = 0.12) and 52% (*p* = 0.05) reductions in MRSA and *P. aeruginosa* biofilm formation, respectively. Further, a 58% (p < 0.01) reduction was demonstrated on micro-patterned surfaces for *P. aeruginosa* biofilms under clinically-simulated conditions when compared to un-patterned controls.

**Conclusions:**

This engineered micro-pattern reduces the colonization and biofilm formation of key VAP-associated pathogens *in vitro*. Future application of this micro-pattern on endotracheal tubes may prevent or prolong the onset of VAP without the need for antimicrobial agents.

## Background

Pneumonia is the most common hospital-acquired infection (HAI) in Intensive Care Units (ICUs)—comprising some 27% of these infections and driving more than half of the antibiotics prescriptions in the ICU [1,2]. Ventilator-associated pneumonia (VAP) is a leading HAI in ICUs, accounting for 86% of nosocomial pneumonia cases [2,3]. The annual U.S. hospital cost of VAP-related treatment is about $1.5 billion and death rates are between 25-60% [4,5]. Approximately 80% of all VAP cases are caused by the ESKAPE pathogens (*Enterococcus faecium, Staphylococcus aureus, Klebsiella pneumoniae, Acinetobacter baumannii, Pseudomonas aeruginosa*, and *Enterobacter* species), which are largely responsible for HAIs and are the primary organisms demonstrating antimicrobial resistance, thereby escaping the effects of antibacterial drugs [6,7]. Current epidemiology shows that *S. aureus, P. aeruginosa, K. pneumoniae, E. coli*, and *A. baumannii* cause 28%, 22%, 10%, 7% and 7%, respectively, of first episode VAP and HAI-associated pneumonia infections world-wide making them the top five causative agents of these infections [7]. Methicillin-resistant *S. aureus* (MRSA) and *P. aeruginosa* comprise the top two causative organisms of VAP and are considered particularly devastating lung pathogens as they cause persistent pneumonia infections, are resistant to a number of antimicrobials, and are associated with a high attributable mortality of patients with VAP [7].

VAP is attributed to the endotracheal tube (ETT) used during ventilation [1,2,5,8]. Intubation does not allow cough reflexes that clear organisms descending from the proximal oropharynx to the distal bronchi [5], therefore the tube provides bacteria access to the lower respiratory tract via contaminated secretions [9]. Also, the surfaces of an ETT provide bacteria with a substratum that promotes microbial colonization and biofilm formation [10-13]. Pathogens isolated from ETT biofilms have been shown to be the same causative organisms responsible for the patient’s VAP, which associates the biofilm presence on the ETT with infection [14,15]. It has been demonstrated that these biofilms can develop on the inner and outer surfaces of the ETT in less than 24 hours of patient intubation [16], and it has been determined that mature biofilm presence rather than duration of intubation is directly related to occurrence of VAP [17].

Treatments for VAP universally include the use of antimicrobials to clear the infection. Prescribing the appropriate antimicrobials at the onset of VAP is increasingly difficult but extremely important, as an inadequate initial antimicrobial therapy is associated with increased mortality [2,18]. Numerous factors contribute to treatment decisions (*e.g.* clinical presentation, local drug resistance patterns, patient exposure) and this complexity prevents the standardization of a VAP antimicrobial treatment guideline [19,20]. Broad-spectrum antimicrobials are often initially implemented to reduce the risk of patient mortality. Unfortunately, this path contributes to increased cost and complications for the patient as well as heightened risk for antimicrobial resistance [2,21]. It is also well known that the presence of antibiotics induces robust biofilm formation of a range of pathogens, which may further contribute to the onset of VAP [22-24]. Common ICU and VAP pathogen drug resistance is steadily rising and outpacing new drug development, leaving healthcare professionals defenseless against these common infections.

To prevent the overuse of anti-microbial treatments, device modifications and improved patient care practices have been implemented to reduce microbial access to the lower respiratory tract. The addition of a subglottal secretion port as well as reducing the thickness of the ETT cuff prevents microaspiration of subglottal secretions into the lungs. These device modifications, in addition to elevation of the patient’s head, daily sedation interruptions, and oral antiseptic treatments, have shown some benefit [5], but the rate of VAP remains substantial. While there is some clinical evidence that silver-coated tubes reduce infection rates [25,26], the cost remains prohibitively high for widespread adoption. A novel solution that would continue to limit bacterial presence in the lower respiratory tract, thereby improving patient outcomes, without promoting antimicrobial resistance is warranted.

**Figure 1:**
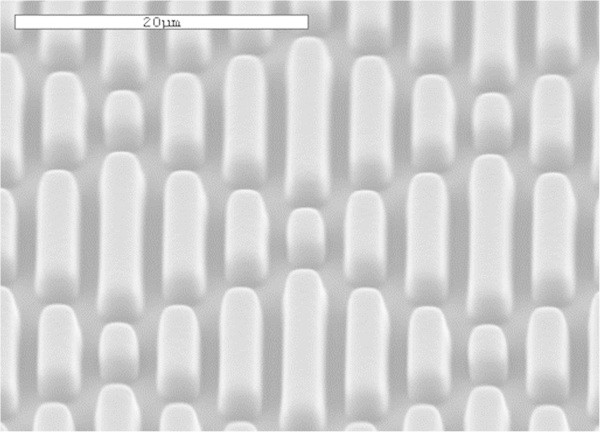
**Scanning electron micrograph of the Sharklet micro-pattern cast into silicone.** This is the micro-pattern used in this study (Scale bar = 20 μm).

Engineered surface topographies, particularly geometries of ordered features designed with unique roughness properties, elicit specific, predictable biological responses and have been shown to control bio-adhesion [27-29]. Studies have shown that the Sharklet micro-pattern (Figure 1) is the most effective among ordered topographies (pillars, channels, other geometries) for inhibiting bio-adhesion [30]. This shark skin-inspired micro-topography may provide an alternative strategy for VAP prevention as it has been shown to inhibit *E. coli* and *S. aureus* biofilm formation *in vitro* without the use of antimicrobial agents [31,32]. The aim of the present study was to determine the effectiveness of this ordered micro-pattern, compared to un-patterned control surfaces, in combating the top five VAP causative pathogens by assessing microbial colonization and biofilm formation on both surface types in simplified and clinically simulated *in vitro* conditions.

## Methods

### Sample fabrication

Micro-patterned-surface samples and un-patterned (control) surface samples were created as described previously [31]. Briefly, silicon wafer molds were created by transferring the Sharklet pattern design to photoresist-coated silicon wafers through photolithography and the features were deep reactive ion etched to a depth of 3 μm before being cleaned and methylated prior to use. Smooth, un-etched silicon wafers were used to create the un-patterned controls. Dow Corning Silastic T-2 poly-(dimethyl siloxane) elastomer films were cast off the silicon wafer molds by mixing one part by weight curing agent with ten parts by weight resin, degassing under vacuum, pressing to 0.4 mm thick with glass plates over the silicon wafer molds, and curing for 1 hour at 65°C. Each flat 0.4 mm thick silicone film was then punched into 12 mm diameter circular samples and tested as described below.

### Bacterial strains, media, and growth conditions

All clinical isolates analyzed in this study were purchased from ATCC (Table 1). These strains include clinically isolated strains of MRSA, *P. aeruginosa*, *K. pneumoniae*, *A. baumannii* and *E. coli*. Two additional strains of *P. aeruginosa* were also assessed to confirm the validity of the results across multiple strains within a species: a second clinical isolate and a laboratory strain. The laboratory *P. aeruginosa* strain PA14, carrying a ∆*bifA* mutation*,* was included in these studies as a comparative strain control due to its genetic modification to overproduce exopolysaccharide and thus form biofilms quickly and consistently [33]. A single colony of each organism, plated on tryptic soy agar (TSA; Criterion), was used to inoculate tryptic soy broth (TSB; Criterion) and grown in a shaking incubator at 37°C and 280 rpm overnight.

**Table 1:** Strains and colonization methodology

**Species**	**Strain Designation**	**Inoculum concentration (CFU/mL)**	**Inoculation duration (hours)**	**Number of replicates per experiment**	**Number of experiments completed**	**Colonization on un-patterned controls (Average log**_**10**_**(CFU) across experiments)**	**Colonization on Micro-patterned samples (Average log**_**10**_**(CFU) across experiments)**	**Repeatability of control colonization (repeatability standard deviation)**
MRSA	ATCC700698	5E + 07	1	3 or 4	3	5.83	3.72	0.05
*P. aeruginosa*	ATCC9027	5E + 06	4	4	3	2.78	1.22	0.53
*P. aeruginosa*	ATCC10197	5E + 06	4	4	4	3.62	2.26	1.94
*P. aeruginosa*	PA14*ΔbifA*	1E + 07	4	4	3	3.96	1.72	0.44
*E. coli*	ATCC700336	5E + 07	4	4	8	3.51	1.21	0.57
*K. pneumoniae*	ATCC27799	5E + 07	4	4	4	4.93	2.01	1.57
*A. baumannii*	ATCC19606	1E + 07	2	4	3	2.74	0.88	0.32

### Colonization assay for the top five VAP-associated pathogens

Micro-patterned and un-patterned control samples were suctioned to the bottom of a sterile Petri dish around the outer perimeter of the dish using ethanol. Overnight microbial cultures were sub-cultured and grown to early log phase in TSB before centrifuging aliquots and resuspending cell pellets with 1x phosphate buffered saline (PBS; CulGeneX). Samples were immersed in inoculum containing ~10^7^ CFU/mL for incubation time of 1 to 4 hours statically at room temperature depending on the propensity of the species to colonize the surface; the cell density on un-patterned controls was targeted to remain within an average of 2.5 to 6 logs (Table 1). After incubation, the inoculum suspension was decanted and samples were rinsed three times by adding sterile 1xPBS to the dish, rotating for 10 seconds on an orbital shaker set to 80 rpm, and decanting the rinsate. The outer edge of each sample was removed to minimize the variability caused by the un-patterned sidewalls by using a sterile 8 mm biopsy punch (VWR International); the 8 mm samples were then aseptically loaded into a 15 mL conical tube containing 2 mL sterile Dey-Engley broth (Sigma Aldrich). Attached cells were removed and disaggregated from each sample surface by vortexing for 30 seconds, sonicating for 2 minutes in a bath sonicator (Branson 3510) and vortexing an additional 30 seconds [34]. Samples were enumerated by a 10-fold dilution series, plating each dilution onto TSA, and counting the colony forming units (CFU) after sufficient growth at 37°C overnight. Each CFU per sample data point was log_10_ transformed before statistical analysis. Each experiment was replicated at least three times for each organism; the exact number of experiments conducted for each organism is shown in Table 1. All experiments, except one MRSA experiment, contained four samples per surface type; that MRSA experiment contained three samples per surface type (Table 1). Statistical analysis took place across the three or more experiments for each organism as described below.

### Biofilm assays

Micro-patterned and un-patterned control samples were suctioned to the bottom of a sterile Petri dish around the outer perimeter of the dish using ethanol. For MRSA biofilms: Overnight cultures of MRSA (ATCC700698) were diluted into fresh TSB to a final bacterial concentration of 10^6^ CFU/mL in TSB. Samples were immersed with 20 mL of the bacterial suspension statically at 37°C for four days, with gentle media replenishing each day from the center of the dish. For *P. aeruginosa* biofilms: Overnight cultures of *P. aeruginosa* ATCC9027 and *P. aeruginosa* PA14Δ*bifA* were diluted into minimal M63 media supplemented with 0.4% arginine (22 mM KH_2_PO_4_, 40 mM K_2_HPO_4_, 15 mM (NH_4_)_2_SO_4_, 1 mM MgSO_4_), or minimal M63 media supplemented with 0.4% arginine, 2 mg/mL porcine mucin that contains the MucA/B complex found in mucus (Sigma Aldrich), and 400 μg/mL oxacillin (Sigma Aldrich), to a final bacterial concentration of 10^6^ CFU/mL [33,35-37]. Samples were immersed with 20 mL of the bacterial suspension statically at 37°C for 24 hours. All dishes were gently rinsed ten times through media exchange by removing 10 mL media from the center of the dish and adding 10 mL sterile 1x PBS. Samples were fixed for 30 minutes in 2.5% gluteraldehyde (Electron Microscopy Sciences) and dehydrated with an ethanol dehydration exchange series (final ethanol concentrations of 25, 50, 75, and 95%). One to three samples were stained with propidium iodide and image stacks were obtained in two to five pre-selected sites per sample by confocal laser scanning microscopy (Zeiss LSM 510 META on Axiovert 200 M). Semi-volumetric analysis was achieved by totaling biofilm area coverage in the x-y plane for each image through the z-stack using ImageJ software after thresholding the image at the brightest frame in the stack [38]. Each biofilm area coverage/image data point was log_10_ transformed before statistical analysis. All biofilm experiments were completed in triplicate.

### Statistical methods

A log reduction (LR) per experiment was calculated by subtracting the average log_10_(micro-pattern data points) from the average log_10_(un-patterned control data points). After confirming the normality of the log reductions by residual and normal probability plots, the mean log reduction was interpreted as the median percent reduction with the equation: 1-10^(− LR)^. Statistical significance of the reductions for the colonization and biofilm assays were assessed using a 1-sided *t*-test of log reductions from at least three experiments per organism. Estimates of the among- and within-experiment variances were assessed using ANOVA of the log transformed cell densities for each un-patterned control sample and micro-patterned sample, with a random effect for experiment. The repeatability standard deviation (SD) of the un-patterned sample, which quantifies the repeatability of the methods based on control variances, is the square root of the sum of the among-experiment variance and the within-experiment variance divided by the number of replicates per experiment [39]. All analyses were performed using the statistical software MiniTab16.

## Results

### Colonization assay for the top five VAP-associated pathogens

**Figure 2:**
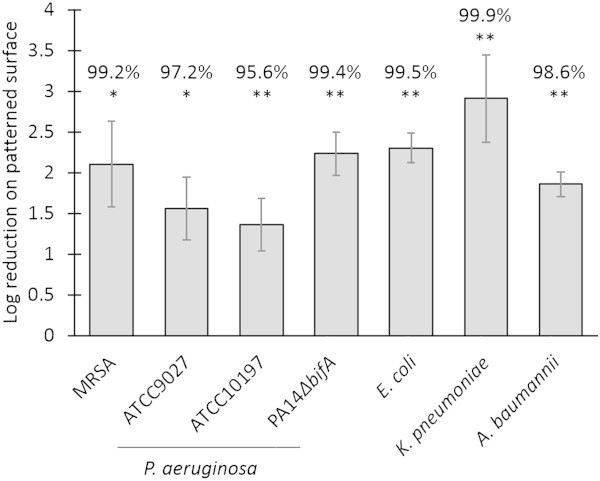
**The micro-patterned surface significantly reduces the colonization of the five microbial species most frequently associated with VAP when compared to un-patterned controls.** All mean log reductions (across at least three experiments) are statistically significantly positive by single *t*-test *p < 0.05; **p ≤ 0.01. Error bars are the standard error of the mean log reduction.

Bacterial colonization on micro-patterned and un-patterned surfaces were evaluated for the top five respiratory pathogens associated with VAP, including clinically isolated strains of MRSA, *P. aeruginosa*, *K. pneumoniae*, *A. baumannii* and *E. coli*. Two additional strains of *P. aeruginosa* were also evaluated to confirm the validity of the results across multiple strains within a species: a second clinical isolate and a laboratory, hyper-biofilm forming strain. The micro-patterned surface significantly reduced the colonization of all species and strains compared to un-patterned controls, with median reductions in CFU counts ranging from 95.6% to 99.9%, *p* < 0.05 when evaluated across multiple experiments (Figure 2). To confirm the consistency of the methods used to obtain these results, the repeatability SD of the log_10_(CFU/control sample) was calculated across experiments for each organism (Table 1). Except for *P. aeruginosa* ATCC10197 and *K. pneumoniae*, all un-patterned repeatability SDs were close to or below 0.50 log, a historical benchmark that indicates highly reproducible colonization on controls across multiple experiments [40]. Even with the increased variability observed for *P. aeruginosa* ATCC10197 and *K. pneumoniae,* the micro-patterned surfaces maintained strong statistically significant performance in this assay thereby indicating good repeatability of the log reductions for all of the organisms tested (Figure 2).

### Biofilm formation of MRSA and *P. aeruginosa* on the micro-pattern

**Figure 3:**
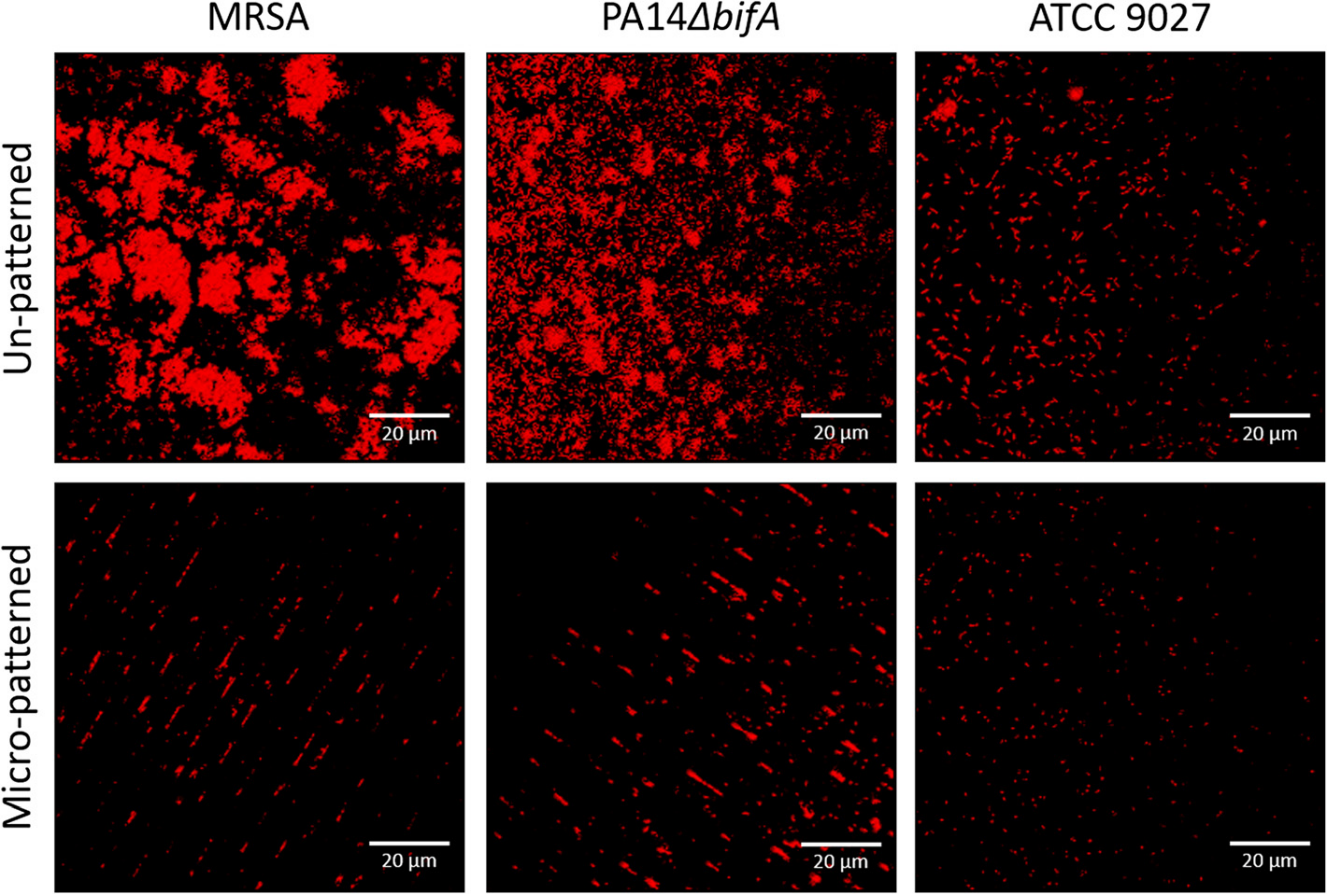
**The micro-pattern reduces biofilm formation of two key VAP-related pathogens, MRSA and *P. aeruginosa *when compared to un-patterned controls.** The *P. aeruginosa* (ATCC 9027) strain did not form robust biofilms under these conditions, however the MRSA and the *P. aeruginosa* PA14ΔbifA strains showed 67% (*p* = 0.123) and 52% (*p* = 0.05) median reductions in biofilm compared to controls, respectively. A representative image per surface type for each organism was selected to reflect quantitative results. Images obtained by compiling the stack of images taken through the biofilm (Scale bar = 20 μm).

Biofilms were grown on micro-patterned and un-patterned surfaces for MRSA and two strains of *P. aeruginosa* followed by confocal imaging and ImageJ analysis to quantify the bacterial area coverage for each image in the z-stacks. The micro-patterned surface showed a 67% (p = 0.123) median reduction of MRSA biofilm volumes grown over a four day duration in a TSB media when compared to un-patterned control surfaces (Figure 3). Similar observations were seen for the hyper-biofilm forming strain *P. aeruginosa* PA14Δ*bifA*, where a significant 52% median reduction in volume (p = 0.05) was observed (Figure 3). Importantly, mature biofilms [17] were obtained on the un-patterned control surfaces for both the MRSA and *P. aeruginosa* PA14*ΔbifA* strains, whereas bacterial colonization on the micro-patterned surfaces was more dispersed (Figure 3). When quantifying the resemblance of the biofilm volume measurements on un-patterned controls, the repeatability SDs of the log-transformed biofilm coverage data was 0.081 for the PA14Δ*bifA* experiments and 0.681 for the MRSA experiments. While the level of acceptable variability of image analysis data has not been established, the small SDs for PA14Δ*bifA* indicates high consistency in biofilm formation on the un-patterned controls. The *P. aeruginosa* ATCC 9027 strain did not form a sufficient quantity of biofilm under these non-clinical conditions to accurately quantify biofilm volume coverage, as most image coordinates had no depth from which to obtain image stacks (Figure 3).

### Biofilm reduction of *P. aeruginosa* in a mucin-modified medium

**Figure 4:**
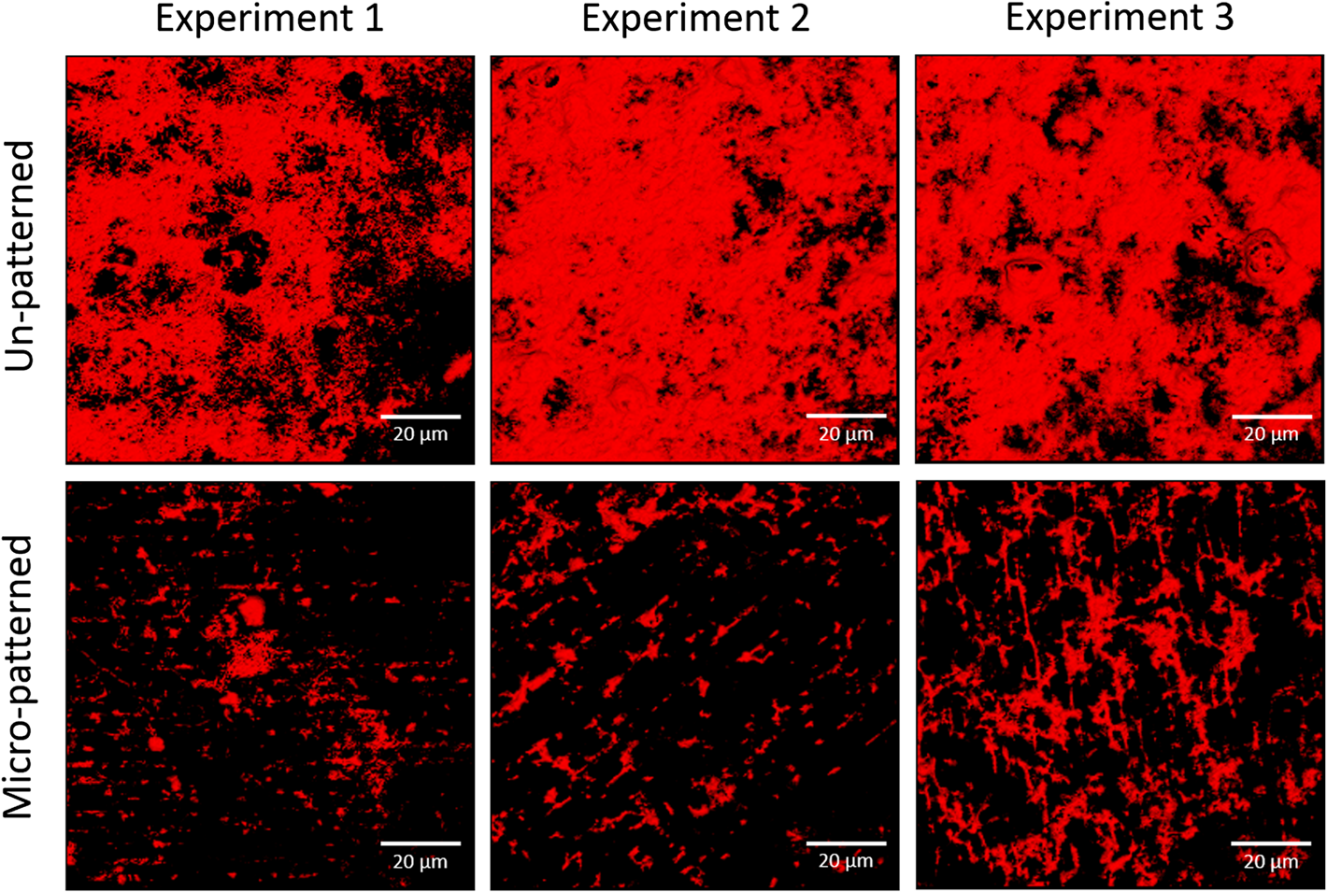
**The micro-pattern significantly reduces *P. aeruginosa *(ATCC 9027) biofilm formation in a mucin-rich environment by an average of 58% (*p*** **= 0.009) over three separate experiments.** A representative image per surface type from each experiment was selected randomly as all images were similar within each experiment. Images obtained by compiling the stack of images taken through the biofilm (Scale bar = 20 μm).

The *P. aeruginosa* clinical isolate ATCC 9027 did not form biofilms in the simplified minimal medium (Figure 3), so additional studies on this strain were conducted in a medium that would both encourage biofilm formation and simulate the clinical environment of the lungs. Thus, a mucin-rich minimal medium supplemented with oxacillin was used to promote biofilm and provide the mucin glycoproteins that make up the main component of the mucus present in the lungs [22-24,35,36]. This medium change resulted in enhanced biofilm formation of this clinical isolate on the un-patterned control surfaces, whereas the micro-patterned surfaces maintained an inhibitory effect, with a 58% reduction compared to controls (p = 0.009) (Figure 4). The repeatability SD for biofilms on controls across three experiments was low at 0.11, suggesting that the methodology used in these assays obtains repeatable biofilm growth and coverage.

## Discussion

Innovative solutions for VAP-prevention via ETT device modifications will be critical for improved patient care, as VAP accounts for 86% of hospital-acquired pneumonia cases in ICUs [2,3]. Recent studies have shown that biofilm development on the surfaces of an ETT is a strong predictor of VAP, with biofilms forming in as little as 24 hours. Current device modifications have had some success in reducing infection rates, particularly cuff modifications that limit microbial access to the lower respiratory tract. However, further limiting microbial access to the lower respiratory tract is crucial for reducing VAP rates in patients. The goal of this study was to determine the feasibility of using a novel micro-patterned surface to decrease microbial colonization for the top five VAP pathogens, and biofilm formation for the top two, under simulated ETT environments.

It is known that surface characteristics modulate microbial interactions – however this is the first study that has shown performance of a specific, ordered micro-pattern against the colonization of the top five organisms that are associated with VAP and other HAIs. Current literature shows that this micro-pattern reduces *S. aureus* and *E. coli* biofilms [31,32], however *P. aeruginosa*, another common biofilm former, had not been tested. This study evaluated the top two causative agents of VAP in biofilm growth assays and showed a reduction of both MRSA and *P. aeruginosa* mature biofilm volume coverage on micro-patterned surfaces when compared to un-patterned controls. Before this study, it was unknown whether biologically relevant proteins, like MucA/B that make up the mucus found in the lungs [36], would coat the micro-pattern and interfere with its capability to reduce microbial colonization. Therefore, *P. aeruginosa* was further challenged in a lung secretion-like mucin medium supplemented with antibiotics, and the micro-pattern demonstrated statistically significant biofilm reduction even under these more challenging conditions. Together, these data contribute to our understanding of the range of bacterial inhibition exhibited by this novel micro-pattern against several clinically relevant pathogens and conditions.

As investigated elsewhere, the theoretical basis for the topography’s function is its ability to create a stable non-wetting state where air pockets at key points in the microscopic features are maintained, thereby inhibiting interaction of microbes with the surface [41]. Specifically, potential attachment sites that would generally induce stable interactions of micro-organisms to the surface are made inaccessible to the organisms due to the non-wetting nature of the topography. This non-wetting property is enhanced by the use of specific nanoforce gradients that occur based on the sizes of the microscopic features, which create an energetically unstable surface to which the micro-organisms are unable to formulate stable attachment modes [42].

A specific growth media formulation and antibiotics were used in this study to achieve clinically simulated biofilm growth *in vitro.* Robust and accelerated *P. aeruginosa* biofilm growth was obtained by using an arginine minimal media to encourage microbial respiration in this biofilm model [43], and to induce a sessile lifestyle that promotes biofilm formation [44]. The nutrient rich mucin protein complex, in addition to antibiotics, have been shown to enhance biofilm formation [22,35,36] and both were included in the medium to induce robust biofilm formation and provide a simulated clinical environment [19]. The combined result was accelerated biofilm formation on control surfaces within 24 hours for *P. aeruginosa* – a goal commonly met by other biofilm reactor systems [45,46].

The biofilms present on un-patterned control surfaces in this study were three-dimensional and made up of large cellular aggregates, which were in contrast to the single cell or small aggregates observed on the micro-patterned surface. Given this observation, implementation of the micro-pattern onto the surface of an ETT could provide a synergistic effect together with the human immune system and antibiotic treatments to clear potentially harmful bacteria before they form a biofilm and cause an infection. Should such a result be demonstrated in a clinical trial, it would ultimately lead to decreased use of antimicrobial treatments and silver-coated ETTs, that may contribute to the rise in antimicrobial resistance observed in the ESKAPE pathogens.

Future work will include demonstrating the efficacy of this micro-pattern in advanced multi-species *in vitro* biofilm models with air-flow and animal VAP models [47-49] utilizing ETT prototypes with the micro-pattern on the inner and outer tube surfaces. These complex models would further our understanding of tissue compatibility and performance of the pattern in clinically relevant environments, before conducting clinical trials that would evaluate patient driven outcomes. Since ventilator-associated complications (VAC) are equally important in prolonging patient recovery [50], future studies will assess the capability of the micro-pattern to reduce tube occlusion, an interesting hypothesis based on the general anti-wetting capability of the micro-patterned surface [51,52]. As this technology has not yet been clinically evaluated, our current results do not indicate what outcomes we can expect for patients who use a micro-patterned ETT. Nonetheless, with future research we hope to develop a micro-patterned ETT that reduces microbial colonization and biofilm formation associated with VAP as well as tube occlusion that leads to VAC. Once demonstrated to be effective in clinical studies, this micro-patterned ETT, combined with state-of-the art device cuff designs and bundle practices, may supply clinicians with an arsenal to diminish VAP and VAC rates.

## Conclusions

The micro-pattern surface modification inhibits the attachment of MRSA*, P. aeruginosa, K. pneumoniae, E. coli,* and *A. baumannii*, the top five VAP-associated pathogens, by up to 99.9% when compared to un-patterned controls. The micro-pattern also inhibits the formation of MRSA and *P. aeruginosa* biofilms, the latter of which was further investigated in a complex mucin-rich medium mimicking the tracheal environment. Demonstration of biofilm reduction by this micro-patterned surface in a range of conditions and with a range of pathogens suggest that the micro-pattern implemented onto ETT surfaces may reduce VAP rates without the use of antimicrobial agents.

## Abbreviations

HAI: Hospital acquired infection; VAP: Ventilator-associated pneumonia; ETT: Endotracheal tube; ICU: Intensive care unit; MRSA: Methicillin-resistant *Staphylococcus aureus*; TSA: Tryptic soy agar; TSB: Tryptic soy broth; PBS: Phosphate buffered saline; SD: Standard deviation; VAC: Ventilator-associated complications.

## Competing interests

Rhea May, Matt Hoffman, Melinda Sogo, and Shravanthi Reddy are employees of Sharklet Technologies, Inc. Al Parker and Anthony Brennan are paid consultants for Sharklet Technologies, Inc.

## Authors’ contributions

RM participated in the design of the study, carrying out the colonization and biofilm assays, statistical analysis, data analysis and interpretation, and drafting the manuscript. MH participated in the design of the study and in carrying out the colonization assays. MS participated in designing and carrying out the colonization and biofilm assays. AP participated in statistical analysis. GO and AB participated in the design of the study and data interpretation. SR conceived the study, participated in its design and coordination, data interpretation, and helped to draft the manuscript. All authors participated in reviewing and editing the manuscript.

## Authors’ information

RM, MH and MS are microbiology researchers and SR is the Director of Research for Sharklet Technologies, Inc. (STI); GO is a biofilm expert and professor at Dartmouth Medical School and an un-paid collaborator with STI; AP is a statistician and professor for the Center of Biofilm Engineering at Montana State University and a consultant for STI; AB is a materials engineer and professor at the University of Florida, Gainesville and is the inventor of the micro-pattern technology where he continues to consult on the technology for STI.
